# New-onset hallucinations with amiodarone: a case report

**DOI:** 10.1186/s12991-022-00409-y

**Published:** 2022-08-24

**Authors:** Jessica Molinaro, Peter DeVries, Jennifer Ha, Jennifer M. Knight

**Affiliations:** 1 Department of Psychiatry and Behavioral Medicine, Medical College of Wisconsin, 8701 Watertown Plank Rd, Milwaukee, WI 53226 USA; 2 Departments of Medicine and Microbiology & Immunology, Medical College of Wisconsin, Milwaukee, WI USA

**Keywords:** Amiodarone, Hallucinations, Case report, Psycho-oncology

## Abstract

**Background:**

Amiodarone is a commonly used antiarrhythmic for the treatment of atrial fibrillation with a unique pharmacokinetic profile. While general side effects can be frequently associated with amiodarone, psychiatric adverse reactions to this medication are uncommon. The relationship between amiodarone and hallucinations independent of delirium has been rarely reported in the literature.

**Case presentation:**

We report the case of a 63-year-old female with a history of estrogen and progesterone receptor positive invasive ductal carcinoma with osseous metastases to the ribs and skull, major depressive disorder, and unspecified anxiety. She was diagnosed with invasive ductal carcinoma 12 years prior and underwent a lumpectomy with axillary lymph node dissection and radiation, currently maintained on anastrozole and trastuzumab for the past 11 years. Her symptoms of major depressive disorder and anxiety have remained in remission on a regimen of bupropion extended release, duloxetine, and trazodone without recent dose changes. This patient presented to the emergency department with dyspnea and was admitted to the general medical floor with new-onset atrial fibrillation. She was subsequently started on amiodarone for rhythm control. Shortly after its initiation, the patient developed new onset auditory and visual hallucinations with an unremarkable extensive medical evaluation. Auditory hallucinations consisted of music and unintelligible conversations, while visual hallucinations were of a family member crying on the floor and a man carrying a gun. The differential diagnoses included depression with psychotic features, delirium, and amiodarone-induced hallucinations. Given the lack of current depressive symptoms, absence of altered cognition, and the temporal relationship between the initiation of amiodarone and the onset of hallucinations, amiodarone was suspected to be probable etiology of her hallucinations. For this reason, amiodarone was replaced with dronedarone. Visual and auditory hallucinations ceased within less than 3 days after the discontinuation of amiodarone.

**Conclusions:**

Psychiatric adverse events from amiodarone are uncommon, and associated isolated hallucinations have only been rarely reported in the literature. While the risk of visual and auditory hallucinations appears to be low with amiodarone initiation, clinicians should be aware of this potential side effect.

## Background

Amiodarone is a Food and Drug Administration (FDA) approved antiarrhythmic medication indicated for ventricular fibrillation or ventricular tachycardia. Although it has not been approved by the FDA for the treatment of atrial fibrillation, it is commonly used for this purpose [1]. Amiodarone prolongs the action potential duration of myocardial cells. It noncompetitively inhibits *α*- and *β*-adrenergic receptors and blocks L-type calcium channels and potassium channels, which produce anti-sympathetic activity and negative chronotropic effects in nodal tissue as well as an increase in the refractory period [2]. Amiodarone has a highly variable bioavailability [1] and has a biphasic elimination with initial one-half decrease of plasma levels after 2.5–10 days. A slower terminal elimination phase shows a mean half-life of the parent compound of 53 days and 61 days for the active metabolite. It is eliminated primarily by hepatic metabolism and biliary excretion [2].

Adverse reactions are common with amiodarone. Pulmonary toxicity, exacerbation of arrhythmia, and hepatic failure are the most serious of side effects^.^ [1] Neurological complaints are common and include fatigue, tremor, poor coordination, ataxia, dizziness, and peripheral neuropathy. These are often dose-related and may improve if the dose is decreased [3]. Amiodarone is an iodine-containing compound with structural similarity to thyroxine; it can cause thyroid abnormalities in about 15% of patients, which can subsequently cause neuropsychiatric symptoms [3, 4]. Psychiatric adverse reactions arising directly from use of amiodarone have been less frequently reported [4]. These have included delirium, catatonic depression, paranoia, confusion, insomnia, agitation, and tiredness [5–9]. Perceptual disturbances have been appreciated in context of delirium associated with amiodarone, however, less frequently outside of delirium, with one case report demonstrating isolated musical hallucinations associated with use of amiodarone without evidence of delirium [10]. We report a case of visual and auditory hallucinations without symptoms of delirium associated with amiodarone use.

## Case presentation

Patient is a 63-year-old female with a past medical history significant for stage IV invasive ductal carcinoma, positive for estrogen receptor (ER), progesterone receptor (PR), and human epidermal growth factor receptor 2 (HER2), with osseous metastases to the calvarium and ribs, heart failure with preserved ejection fraction, diabetes mellitus type 2, hypertension, obstructive sleep apnea, unspecified anxiety disorder, and recurrent major depressive disorder. The patient did not have metastases to her brain. She was diagnosed with metastatic breast cancer 11 years prior and has undergone lumpectomy with lymph node dissection and radiation therapy. The patient had been maintained on chemotherapy with anastrozole and trastuzumab without any evidence of disease progression. Patient was admitted to the general medical floor for new onset atrial fibrillation with rapid ventricular response after experiencing worsening dyspnea in the 2 days preceding hospitalization.

On admission, she was placed on adenosine and diltiazem infusions for her continued tachycardia. Despite these medications, the patient remained in atrial fibrillation. The patient underwent a thorough medical work-up, which was overall unremarkable other than a transthoracic echocardiogram, which showed new left ventricular dysfunction with an ejection fraction of 36–40%. Trastuzumab was subsequently discontinued in context of newly noted reduced left ventricular function. She then underwent successful transesophageal echocardiogram with direct current cardioversion to normal sinus rhythm. During this hospitalization, she did not demonstrate any evidence of altered mentation or psychiatric symptoms. The patient remained in normal sinus rhythm and was placed on amiodarone 200 mg twice daily and dabigatran 150 mg twice daily. No additional medication changes were made prior to discharge from hospital.

Days after hospital discharge, this patient began to experience dizziness and fatigue. In addition, she developed hallucinations about 7 days after having been started on amiodarone. She endorsed two vivid visual hallucinations, which included seeing a man with a gun in her hallway and her sister-in-law crying on the floor. She also endorsed auditory hallucinations of unintelligible conversations and music. She did not have any changes in attention, cognition, or executive function that would indicate signs of delirium. On further psychiatric review of systems, she did not endorse symptoms of anxiety, mood disturbance, or substance use. The patient had no prior history of hallucinations or other psychotic symptoms. Her psychiatric history was significant only for symptoms of depression and anxiety, which initially presented in the context of her cancer diagnosis 12 years prior and had been well managed on duloxetine 60 mg, bupropion extended release 300 mg, and trazodone 50 mg at bedtime. She was subsequently assessed in the emergency department, where a medical evaluation, including comprehensive metabolic panel, complete blood count, thyroid-stimulating hormone, urinalysis, and computed tomography of head, was unrevealing. Given the temporal association of the onset of her hallucinations with the initiation of amiodarone, there was concern that the hallucinations were related to this medication. After discussion between the psychiatry and cardiology teams, amiodarone was discontinued and dronedarone was started. No other medication changes were made at this time. Within 2–3 days of discontinuing the amiodarone, her visual and auditory hallucinations completely resolved. Upon continued and routine psychiatric follow-up approximately 6 months after cessation of amiodarone, the patient continued to present with stability of mood and anxiety symptoms and no further recurrence of hallucinations.

## Conclusions and discussion

We report on a patient with invasive ductal carcinoma with osseous metastases to the calvarium and ribs, major depressive disorder and unspecified anxiety who developed disturbing visual and auditory hallucinations after starting amiodarone for new-onset atrial fibrillation. As the hallucinations emerged shortly after the initiation of amiodarone and remitted with its discontinuation, we suspect that these hallucinations were associated with amiodarone use. To help establish the relationship between the use of amiodarone and subsequent hallucinations, the Adverse Drug Reaction Probability Scale, which is also known as the Naranjo Algorithm, was utilized. Total scores range from −4 to  + 13; the reaction is considered definite if the score is 9 or higher, probable if 5–8, possible if 1–4, and doubtful if 0 or less [11]. Per the Naranjo Algorithm, we found that this patient scored a “5” which indicates a probable association between her hallucinations and amiodarone. Points earned to support this association include her hallucinations appeared after receiving amiodarone (+2), resolved with its discontinuation (+1), and that no alternative causes on their own were identified to have caused the reaction (+2).

Alternative etiologies to amiodarone for the patient’s hallucinations were considered. Hallucinations can both be seen in psychosis and in context of substance intoxication and withdrawal. These etiologies appear less likely as our patient has no history of a primary psychotic disorder nor substance abuse. Review of her history was notable for one instance of hallucinations about 2 years prior to current episode that was thought to be in context of delirium as it presented when patient had influenza. Her current medications were also reviewed to evaluate for potentially contributing to hallucinations. Medications that she was taking with known risk for neuropsychiatric side effects included bupropion extended release [12], primidone [13], and topiramate [14]. Anastrozole has also been linked to hallucinations in case reports [15]. These medications are all longstanding and without any recent dose modifications during this episode that correlated with the emergence or resolution of patient’s hallucinations, suggesting this to be a less likely etiology. Review of medications administered during her hospital course was also notable for being started on a diltiazem infusion for about 24 h on the first day of her hospitalization. Case reports linking diltiazem with psychosis and an episode of secondary mania were noted in the literature [16]; however, this appears less likely to be the underlying etiology given emergence of hallucinations greater than 1 week after diltiazem was discontinued.

Finally, a thorough medical work-up was conducted during evaluation of hallucinations that did not suggest underlying etiologies related to infection, metabolic derangements, thyroid dysfunction, or a traumatic intracranial lesion. No clinical findings to suggest evidence of delirium. While it would have been ideal to have a urinary or serum drug screen obtained to conclusively rule out any contribution from a substance-induced etiology, patient had no history of substance misuse, denied any current substance misuse, and the temporal link of emergence and resolution of hallucinations coincided with trial and discontinuation of amiodarone. In context of this temporal link of her symptoms with use of amiodarone and the other considered etiologies appearing less likely, it appears that patient’s hallucinations are associated with amiodarone use. Other antiarrhythmic agents have been associated with neuropsychiatric side effects.in case reports. Digoxin, another class III antiarrhythmic agent, has been associated with psychiatric symptoms, including psychosis, both at therapeutic levels and in toxicity. Most class I antiarrhythmic agents, including procainamide, quinidine, lidocaine, and flecainide, have also been associated with symptoms of psychosis in case reports [4]. Reemergence of hallucinations with a rechallenge with amiodarone would have helped in establishing this association more definitively. A rechallenge was avoided as patient also experienced dizziness and bradycardia with use of amiodarone, and cardiology recommended using an alternate antiarrhythmic agent, dronedarone, in its place. To the best of our knowledge, isolated hallucinations associated with amiodarone, without signs of delirium or other psychiatric symptoms, have been rarely reported in the literature [15].

While neurologic side effects, including dizziness, tremor, and ataxia, are common with amiodarone, psychiatric adverse reactions of amiodarone are infrequent [3]. Hallucinations associated with amiodarone have been previously reported in the context of delirium [6] or co-occurring with other psychiatric symptoms [9, 17]. This patient is notable for developing new-onset, isolated perceptual disturbances with amiodarone. Interestingly, a similar case was reported in the literature related to onset of musical hallucinations with amiodarone use and no evidence of delirium. Our patient also endorsed musical hallucinations in addition to other auditory and visual hallucinations. These hallucinations emerged within 1 week of starting amiodarone therapy and ceased within 3 days of discontinuation. Barry et al. [6] reported a case in which symptoms of delirium appeared 4 days after starting amiodarone, improved rapidly with its discontinuation, and remerged after 4 days of re-challenge. Other case reports have indicated onset of psychiatric symptoms, including delirium and depression, within several days [18] to weeks [7, 9, 17] of initiating amiodarone. Many of these studies have demonstrated relatively rapid and dramatic improvement within days of its discontinuation [5, 7, 18, 19].

This variability in time to onset of psychiatric symptoms after starting amiodarone is likely secondary to its unique pharmacokinetic profile. Amiodarone is notable for its highly variable absorption, oral bioavailability, and volume of distribution. With its extensive accumulation in various sites including adipose tissue and highly perfused organs, such as the liver, lung and spleen, amiodarone has a large volume of distribution [3]. Taken together, these properties contribute to a variable onset of action, ranging from several days to a few weeks, in addition to a long elimination half-life of approximately 58 days following single dose administration [2] The relatively rapid improvement of psychiatric adverse effects after discontinuation of amiodarone seen in our patient and in multiple other case reports can likely be explained by amiodarone’s biphasic elimination properties. The initial elimination phase consists of a 50% reduction of plasma amiodarone levels after 2.5–10 days, reflecting its elimination from well-perfused tissue. This is followed by a second and slower terminal plasma-elimination phase, with a mean elimination half-life of approximately 53 days for amiodarone and 61 days for its active metabolite, N-desethylamiodarone for those patients on chronic oral therapy [2].

In conclusion, amiodarone is a commonly encountered medication with unique pharmacokinetic properties and a highly diverse side effect profile. It is important for psychiatrists to be aware of the potential for uncommon psychiatric adverse effects, including new-onset auditory and visual hallucinations, and the variability in timing of these episodes as was demonstrated in the current case. Additional research is warranted to further appreciate the risk of psychiatric adverse reactions from amiodarone.

## Data Availability

Data sharing is not applicable to this article as no data sets were generated or analyzed during the current study.

## Abbreviations

FDA: Food and Drug Administration
ER: Estrogen receptor
PR: Progesterone receptor
HER2: Human epidermal growth factor receptor 2
